# Comparison of intramedullary myeloma and corresponding extramedullary soft tissue plasmacytomas using genetic mutational panel analyses

**DOI:** 10.1038/bcj.2016.35

**Published:** 2016-05-20

**Authors:** S J de Haart, S M Willems, T Mutis, M J Koudijs, M T van Blokland, H M Lokhorst, R A de Weger, M C Minnema

**Affiliations:** 1 Department of Clinical Chemistry and Hematology, UMC Utrecht, Utrecht, The Netherlands; 2 Department of Molecular Pathology, UMC Utrecht, Utrecht, The Netherlands; 3 Department of Hematology, VU Medical Center, Amsterdam, The Netherlands; 4 Medical Genetics, UMC Utrecht, Utrecht, The Netherlands; 5 Department of Hematology, Cancer Center UMC Utrecht, Utrecht, The Netherlands

Multiple Myeloma (MM) is characterized by growth and proliferation of clonal plasma cells in the bone marrow (BM), and these plasma cells depend heavily on this BM microenvironment. In a subset of patients, the myeloma cells can spread hematogenously to soft tissues and these extramedullary myeloma (EM) lesions may be found in up to 30% of MM patients, the majority occurring during the course of the disease.^1^ When restricting the definition of EM lesions to infiltration of soft tissue only and excluding the bone-related plasmacytomas, the prognosis is dismal with an overall survival of less than 6 months.^1, 2^ It has been suggested that the incidence of EM relapses is increasing, possibly due to novel treatments or allogeneic stem cell transplantation.^3^ Therefore novel therapeutic interventions are warranted. Molecular and DNA sequencing studies have revealed that MM is a genetically complex and heterogeneous disease,^4, 5^ which demonstrated that molecular events in MM are not attained in a linear manner, but show intraclonal heterogeneity. Each subclone may carry novel mutations, which can influence drug sensitivity. Possibly due to these impediments, little progress had been made thus far in specific therapeutic targeting of oncogenic mutations in MM. An illustrative example of a successful targeted therapy is the treatment of a MM patient carrying a BRAFV600 mutation and EM disease with BRAF inhibitor vermurafenib.^6^ However, the frequency of these BRAFV600E mutations is low: around 8.5% in EM and around 4% in general MM population.^5, 6^ Another pitfall in the development of more effective treatment for relapsed EM patients is the lack of knowledge of the exact pathogenic or molecular mechanisms of the transition to EM and the independency of the BM. One of the suggested mutations involved in this transition is RAS mutations, since RAS mutations were found in three out of six EM samples, which were not detectable in the matched BM samples.^7^

To screen for specific alterations between primary MM BM biopsies and EM relapses with the aspiration to identify possible novel therapeutic targets, we sequenced DNA of a well-documented cohort of MM patients with EM disease for a targeted panel of 50 tumor suppressor and oncogenes, often mutated in cancer.

We selected 14 MM patients diagnosed at our institution from 2000 till 2015 from whom both formalin-fixed, paraffin-embedded material of soft tissue EM relapse and a BM biopsy at diagnosis were present. Clinical data and cytogenetic data (karyotyping and FISH) were retrieved from databases and medical records. The study was approved by the Scientific Advisory Board Biobanking of the University Medical Center Utrecht.

A pathologist confirmed MM diagnosis and demarcated the tumor area. To obtain a high percentage of tumor cells, only samples with at least 10% tumor cells were selected for further processing and to enrich for tumor cells they were dissected with a scalpel from the biopsy material using 10 mesodissected 4-μm-thick paraffin sections. DNA of dissected tumor cells was isolated by DNA sample preparation kit (Roche, Basel, Switzerland) according to the manufacturer's protocol. Next generation sequencing was performed on the IonTorrent PGM using AmpliSeq Cancer Hotspot V2 Panel. This panel primarily contains amplicons to detect currently known cancer-associated mutations in the following actionable cancer genes: ABL1, AKT1, ALK, APC, ATM, BRAF, CDH1, CDKN2A, CSF1R, CTNNB1, EGFR, ERBB2, ERBB4, EZH2, FBXW7, FGFR1, FGFR2, FGFR3, FLT3, GNA11, GNAS, GNAQ, HNF1A, HRAS, IDH1, IDH2, JAK2, JAK3, KDR, KIT, KRAS, MET, MLH1, MPL, NOTCH1, NPM1, NRAS, PDGFRA, PIK3CA, PTEN, PTPN11, RB1, RET, SMAD4, SMARCB1, SMO, SRC, STK11, TP53 and VHL. The samples were processed according to the manufacturer's protocol and statistics were performed as previously described.^8^ Besides genetic analysis, we also performed immunohistochemical (IHC) analysis for p53 protein expression as previously described.^9^

The characteristics of the patients included in the analysis are shown in Table 1. All patients had been treated with immunomodulatory drugs and 10 with a proteasome inhibitor. In total, 7 out of 14 patients developed the EM relapse after allogeneic stem cell transplantation and/or donor lymphocyte infusion. In total, 12 out of 15 BM biopsies yielded results and 11 out of 14 EM biopsies, demonstrating a success percentage of DNA retrieval in 80% and 79%, respectively. The EM biopsies that were analyzed were located in the lymph node (2), skin (7), orbita (1) and pancreas (1).

Overall a limited number of mutations was found in these samples (Figure 1), most samples containing only a single mutation, with a maximum of three mutations in one sample. Somatic mutations were found in NRAS, KRAS, Kit c840, ATM, PAC, TP53 and BRAF.

A high prevalence of activating RAS mutations was found both in BM samples in 6 out of 9 patients (67%) and in EM samples in 7 out of 11patients (64%). The frequency of RAS mutations in this cohort is much higher than the previously reported frequencies of 23–44% in newly diagnosed and relapsed MM patients^10, 11^ and is in accordance with the high incidence of RAS mutations reported in plasmacell leukemias from 54.4% at diagnosis to 81% at time of relapse.^12^ In five patients with an RAS mutation, we were able to compare the BM and EM samples and demonstrated that in three patients the identical RAS mutation was already present in the diagnostic BM samples. In one patient it was not detectable in the BM at diagnosis, but was present in the BM at relapse and only one patient had a gain of RAS mutation in the EM sample only. This contradicts previous findings that the mutation is acquired during the disease progression from intramedullary to EM disease.^7^ The high frequency of RAS mutations does support, however, the previous hypothesis that RAS might be a prerequisite for EM growth,^7^ next to a yet-unidentified mechanism.

Further focusing on the seven patients with paired BM and EM analysis available in our cohort, we found no additional mutations that are likely to be causal for EM spread, nor did we find novel treatment targets using this limited gene panel. We did observe an increment in allele frequency in some cases for mutations present in EM compared with BM, which may be due to the clonal selection, however this may also be attributed to the presence of non-tumor cells in the biopsies.

In our cohort, 1 out of 11 patients had the BRAFV600E mutation in the EM biopsy, which coincide with previously reported prevalence of BRAF mutation in EM.^6^ Furthermore, we detected three mutations, not previously described in MM patients: KIT C840Y/C844Y, ATM L2877F and APC E1317Q. Mutant KIT has been implicated in the pathogenesis of several cancers including melanoma, acute leukemia and gastrointestinal stromal tumors, but effective treatment strategies are still missing. ATM mutations are commonly found in ataxia-telangiectasia and somatic mutations were recently found to be related to the loss of the 11q23 region in T-PLL, however this is not commonly found in MM. These novel mutations in MM were both found in BM material at diagnosis and in EM tissue, and are therefore unlikely to be related to the development of EM disease. Furthermore, no healthy control tissue of these patients was analyzed and therefore it is not certain that these mutations are involved in the pathogenesis of MM.

TP53 mutations may be associated with the presence of EM disease at diagnosis.^10^ In three patients, a TP53 mutation or frameshift was detected in their BM or EM relapse sample. These three patients all showed diffuse and strong nuclear expression of the p53 protein on IHC, also indicative for a TP53 mutation (data not shown). Our IHC analyses also revealed a p53 overexpression in the EM relapse of two patients that did not have a mutation in TP53 and their BM samples had normal and overexpression of TP53, respectively. This is consistent with the general understanding that TP53 mutations are rarely present at the time of diagnosis but occur more frequently in advanced disease as well as in EM disease.^13^ We should note, however, that in patient number 1, the TP53 mutation was detected in the BM relapse but not the EM relapse. This indicates that clones with TP53 aberrations do not necessarily have to persist or disseminate in EM disease.

Interestingly, the TP53V197L mutation found in the EM relapse of patient 13 is associated with resistance to radiotherapy (RT) in patients with solid malignancies like glioblastoma, and head and neck cancer.^14^ The EM plasmacytoma of this patient was also RT refractory suggesting, for the first time, that the outcome of RT may also be linked to TP53 mutations in MM.

In conclusion, we demonstrate the feasibility of performing next generation sequencing on formalin and decalcified BM biopsy material of MM patients. Patients with an EM relapse have a high frequency of 69% of RAS mutations, in most of them already present at diagnosis. The frequency of TP53 mutations is less and mostly detected in relapsed samples. Therefore, the typical behavior and therapy resistance of EM relapse seems to be mediated by other factors than analyzed in this study and are possibly related to post-transcriptional alterations and/or to the influence of the micro environment.^15^

## Figures and Tables

**Figure 1:**
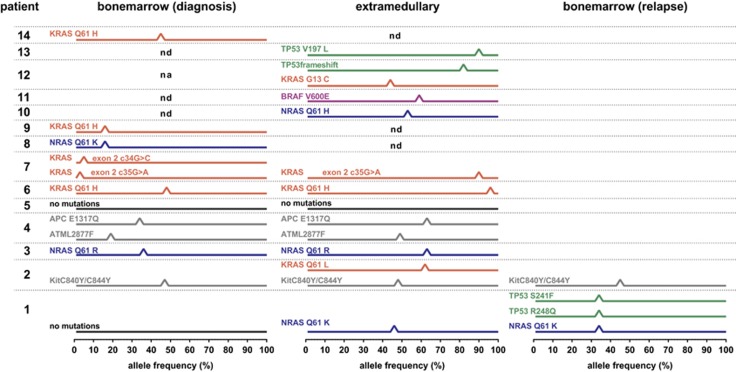
Schematic representation of somatic mutations identified in 14 multiple myeloma patients. The mutant allele frequency of each mutation observed on the IonTorrent PGM using AmpliSeq Cancer Hotspot V2 Panel was plotted for each individual patient indicated, in which sample the mutation was found. ND, not determined, due to technical issues NA, not applicable, unable to retrieve sample for analysis.

**Table 1:** Patient characteristics (*n*=14)

*Patient no*	*Gender*	*Age at diagnosis*	*Ig-type*	*% PC in diagnostic BM*	*Lines of therapy before EM*	*EM years after diagnosis*	*Cytogenetics*	*Allogeneic SCT*
1	F	46	IgGλ	60%	5	7	48 XX FISH:nd	Yes
2	F	49	IgGκ	40%	10	11	56 XX (hyperdiploid) FISH: no deletions	Yes
3	M	35	Light chain	35%	2	1	FISH: 1q+	No
4	F	63	Light chain	ND	2	1	FISH: 1q+	Yes
5	M	30	IgGκ	NA	0	0	ND	No
6	M	61	IgAλ	90%	2	0.5	ND	No
7	M	49	IgGκ	40%	7	5	Hyperdiploid FISH: 1q+	Yes
8	F	69	IgGκ	70%	2	2	46 XX Fish del 13q14, trisomy 9	No
9	M	64	Light chain	90%	1	1	46 XY FISH: no deletions	No
10	M	52	IgAκ	NA	5	4	ND	Yes
11	M	69	IgGλ	NA	2	1	ND	No
12	M	43	IgGκ	NA	9	10	56-56 XY (hyperdiploid) FISH: no deletions	Yes
13	M	55	IgGκ	85%	6	6	ND	Yes
14	F	39	IgGλ	40%	0	0	Near tetraploid FISH: no deletions	No

Abbreviations: EM, extramedullary; NA, not applicable; ND, not determined; PC, plasma cells; SCT, stem cell transplantation.
